# Microbial diversity and biogeochemical cycling in soda lakes

**DOI:** 10.1007/s00792-014-0670-9

**Published:** 2014-08-26

**Authors:** Dimitry Y. Sorokin, Tom Berben, Emily Denise Melton, Lex Overmars, Charlotte D. Vavourakis, Gerard Muyzer

**Affiliations:** 1 Winogradsky Institute of Microbiology, RAS, Moscow, Russia; 2 Department of Biotechnology, Delft University of Technology, Delft, The Netherlands; 3 Institute for Biodiversity and Ecosystem Dynamics, University of Amsterdam, Amsterdam, The Netherlands

**Keywords:** Biogeochemical cycling, Haloalkaliphile, Halophile, Meta-omics, Soda lake, Systems biology

## Abstract

Soda lakes contain high concentrations of sodium carbonates resulting in a stable elevated pH, which provide a unique habitat to a rich diversity of haloalkaliphilic bacteria and archaea. Both cultivation-dependent and -independent methods have aided the identification of key processes and genes in the microbially mediated carbon, nitrogen, and sulfur biogeochemical cycles in soda lakes. In order to survive in this extreme environment, haloalkaliphiles have developed various bioenergetic and structural adaptations to maintain pH homeostasis and intracellular osmotic pressure. The cultivation of a handful of strains has led to the isolation of a number of extremozymes, which allow the cell to perform enzymatic reactions at these extreme conditions. These enzymes potentially contribute to biotechnological applications. In addition, microbial species active in the sulfur cycle can be used for sulfur remediation purposes. Future research should combine both innovative culture methods and state-of-the-art ‘meta-omic’ techniques to gain a comprehensive understanding of the microbes that flourish in these extreme environments and the processes they mediate. Coupling the biogeochemical C, N, and S cycles and identifying where each process takes place on a spatial and temporal scale could unravel the interspecies relationships and thereby reveal more about the ecosystem dynamics of these enigmatic extreme environments.

## Introduction

**Fig. 1:**
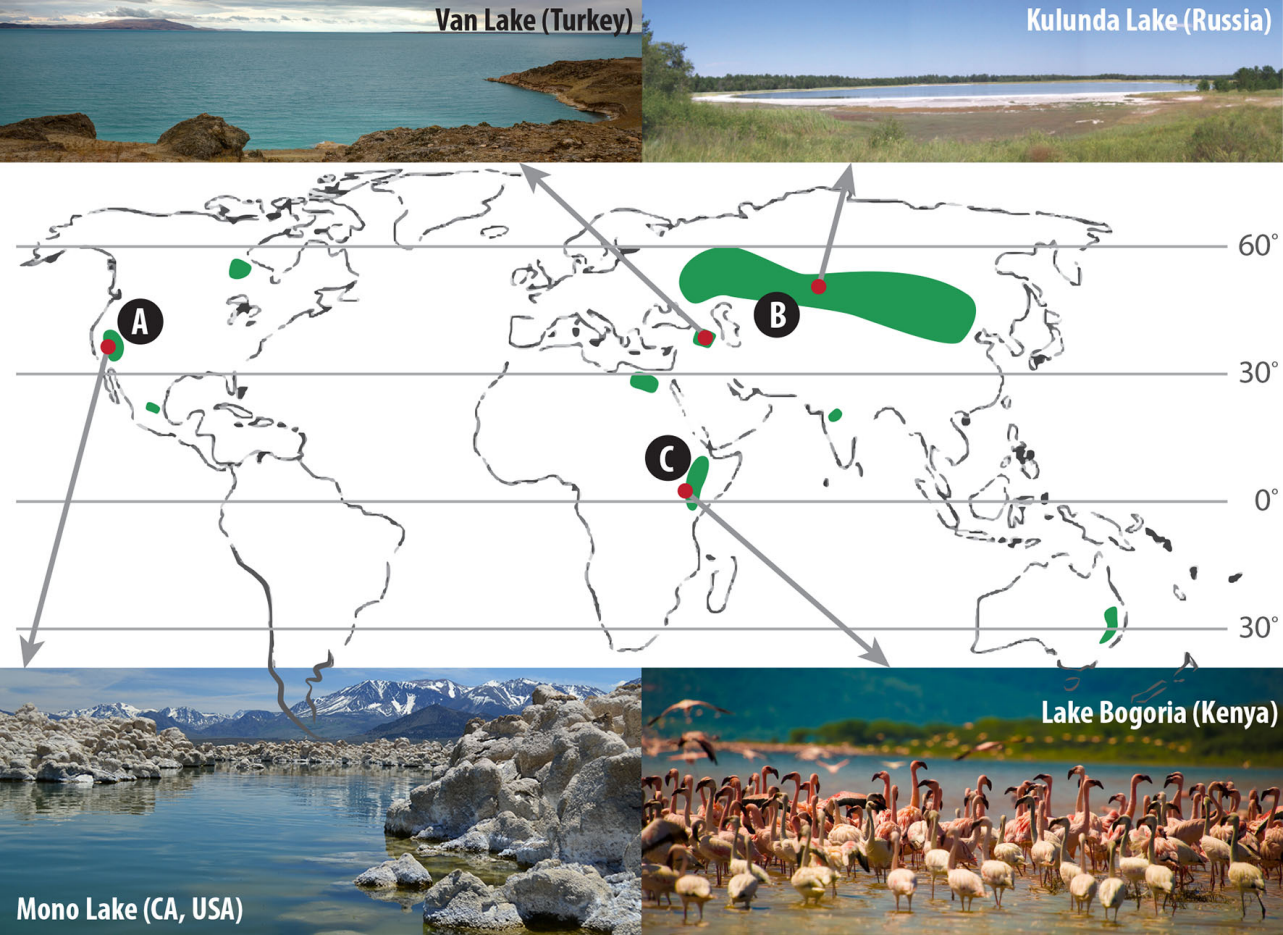
World map depicting major areas where soda lakes occur (*green*).* A* Rain shadowed area of California and Nevada. Mono Lake is depicted (photograph by Sacha Heath).* B* Eurasian Steppe contains the Kulunda steppe and Kulunda Lake.* C* Rift Valley contains many soda lakes, such as Lake Bogoria (photograph from Shutterstock). Shown in the *top left* is Van Lake in Turkey (photograph from Shutterstock). Also indicated are the Central Mexican plateau, Manitoba (Canada), Wadi al Natrun (Egypt), Decan Plateau (India), and Eastern Australia

Soda lakes are found worldwide, predominantly in arid and semi-arid environments, such as the Rift Valley in East Africa, the rain-shadowed regions of California and Nevada, and the Kulunda Steppe in South Siberia (Russia) (Fig. 1). Soda lakes are formed in depressions where ground water rich in carbon dioxide, but poor in magnesium and calcium, leaches sodium from sodium-rich rocks. The absence of dissolved divalent cations is crucial to avoid carbonate precipitation. During arid climate conditions in closed basins, carbonate salts become more concentrated due to increased evaporation rates, leading to the formation of natural sodium carbonate/bicarbonate-buffered systems with elevated pH values (9.5–11) and salt concentrations up to saturation (Tindall 1988; Grant et al. 1990). The chemical composition of the prevailing salts leads to perfect conditions for haloalkaliphiles to thrive. Natronophily indicates a preference for sodium carbonates over sodium chloride, the dominant salt in thalassic (hyper)saline environments, and is based on the fundamental difference in the electrolytic and osmotic properties of these two sodium salts (Banciu et al. 2004; Banciu and Sorokin 2013). Low and moderately saline soda lakes (total salinity between 35 and 50 g/L and 50 and 250 g/L, respectively) are highly productive and harbor fully functional and diverse haloalkaliphilic microbial communities responsible for the cycling of chemical elements, such as carbon, nitrogen, and sulfur. Under hypersaline conditions (total salinity >250 g/L) the diversity is restricted to a few extremely salt-tolerant specialists (Ochsenreiter et al. 2002; Mesbah et al. 2007). The carbon and nitrogen cycles are presumably partly inhibited, as follows from the lack of cultured methanotrops at moderate salinity (Sorokin et al. 2000; Trotsenko and Khmelenina 2002) and the cessation of nitrification at high salt concentrations (Sorokin 1998).

Soda lakes are ‘treasure troves’ for biotechnologists, because they harbor extremophiles with the potential to produce enzymes (extremozymes) that are active both at a high pH and high salinity. Alkali-stable extracellular proteases, lipases, and cellulases have been used for the production of improved laundry detergents (Horikoshi 2006). Halo-alkali-stable cellulases can also be used to release sugars from recalcitrant lignocellulose in agricultural waste for the production of bioethanol. These enzymes have an additional advantage, because ionic liquids (organic analogues of inorganic salts) are frequently used during pretreatment in the solubilization of (ligno) cellulosic biomass (Zhu 2008; Zavrel et al. 2009; Zhang et al. 2011). Besides the discovery of novel hydrolases, a novel nitrile hydratase was isolated from the soda lake Actinobacterium *Nitriliruptor alkaliphilus* (van Pelt et al. 2008; Sorokin et al. 2009). Nitrile hydratases are important industrial enzymes that catalyze the hydration of a broad scope of nitrile compounds into commercially more valuable amides (e.g. acrylamide). Apart from these extremozymes, whole cells of haloalkaliphiles can be used for the sustainable removal of toxic sulfur compounds from wastewater (Janssen et al. 2009; de Graaff et al. 2011) and gas streams (van den Bosch et al. 2007; Sorokin et al. 2008f; Janssen et al. 2009), and for the biodegradation of hydrocarbons and other organic (e.g. nitro-aromatics) and inorganic (e.g. arsenic, uranium) pollutants (Sorokin et al. 2012c).

**Fig. 2:**
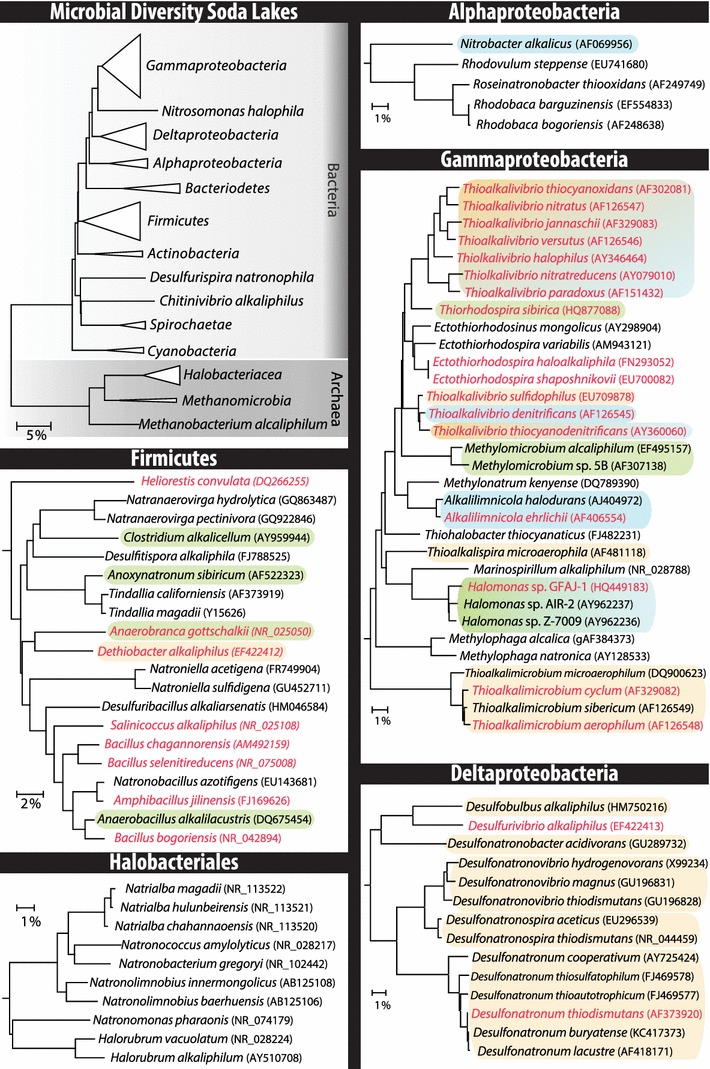
Phylogenetic tree of identified bacteria and archaea in soda lakes. Indicated are the cultured microbes whom have been shown to be active in biogeochemical cycling (*green* carbon cycle, *blue* nitrogen cycle, *yellow* sulfur cycle, see Fig. 3). The *red* font indicates that the genome of the strain has been sequenced

Here we present an overview of the cultured (Fig. 2 and Table 1) and uncultured bacterial and archaeal diversity of soda lakes and focus on the role of these microorganisms in the biogeochemical carbon, nitrogen, and sulfur cycles. In addition, we discuss the molecular mechanisms that allow these haloalkaliphilic prokaryotes to thrive at the double-extreme conditions of high pH and high salinity.

**Table 1:** Microbial species isolated from soda lakes

Genus	Species	Sample origin	Source	Genome	Size (Kb)	Gene-count	GC (%)
*Thioalkalivibrio*	*Tv. denitrificans*	Lake Bogoria (Kenya)	Sorokin et al. (2001c)	NA			
	*Tv. jannaschii*	Mono Lake (CA, US)	Sorokin et al. (2002a)	NA			
	*Tv. versutus*	Siberia (Tuva region)	Sorokin et al. (2001a)	PD	5844	5597	66
	*Tv. nitratis*	Lake Nakuru (Kenya)	Sorokin et al. (2001a)	NA			
	*Tv. thiocyanoxidans*	Kulunda Steppe (Siberia, Russia)	Sorokin et al. (2002b)	PD	2765	2677	66
	*Tv. paradoxus*	Kenya/Wadi Natrun (Egypt)	Sorokin et al. (2002b)	PD	3364	3233	67
	*Tv. nitratireducens*	Lake Fazda (Wadi Natrun, Egypt)	Sorokin et al. (2003)	Complete	4002	3875	66
	*Tv. thiocyanodenitrificans*	Wadi Natrun (Egypt)/Kulunda steppe (Siberia, Russia)	Sorokin et al. (2004b)	PD	3747	3679	65
	*Tv. halophilus*	Stamp Lake (Kulunda Steppe, Russia)	Banciu et al. (2004)				
*Thioalkalimicrobium*	*Tm. cyclicum*	Mono Lake (CA, US)	Sorokin et al. (2002a)	Complete	1932	1734	47
	*Tm. aerophilum*	East African Rift Valley (Kenya)/Siberia (Russia)	Sorokin et al. (2001a)	Complete	2158	2111	46
	*Tm. sibericum*	Siberia (Russia)	Sorokin et al. (2001a)	NA			
	*Tm. microaerophilum*	Soap Lake (WA, USA)	Sorokin et al. (2007a)	NA			
*Thioalkalibacter*	*Ta. halophilus*	Kulunda Steppe (Siberia, Russia)	Banciu et al. (2008)	NA			
*Thioalkalispira*	*Ts. microaerophila*	Lake Fazda (Wadi Natrun, Egypt)	Sorokin et al. (2002c)	NA			
*Ectothiorhodospira*	*Es. variabilis*	Lake Um-Risha (Wadi Natrun, Egypt)	Gorlenko et al. (2009)	NA			
	*Es. vacuolata*	L. Bogoria, L. Nakuru, L. Elmentieta, Crater Lake, L. Magadi (Kenya)	Imhoff et al. (1981)	NA			
*Thiorhodospira*	*Tr. sibirica*	Malyi Kasytui (Siberia, Russia)	Bryantseva et al. (1999)	NA			
*Ectothiorhodosinus*	*Ers. mongolicus*	Dzun Uldziin Nur (Mongolia)	Gorlenko et al. (2004)	NA			
*Desulfonatronum*	*Dn. thiodismutans*	Mono Lake (CA, US)	Pikuta et al. (2003a)	IP	NA	NA	63
	*Dn. lacustre*	Lake Khadyn (Tuva Region, Siberia, Russia)	Pikuta et al. (1998)	PD	3791	3460	59
	*Dn. thioautotrophicum*	Tanatar-1 (Kulunda Steppe, Siberia, Russia)	Sorokin et al. (2011c)	NA			
	*Dn. thiosulfatophilum*	Picturesque (Kulunda Steppe, Siberia, Russia)	Sorokin et al. (2011c)	NA			
*Desulfonatronovibrio*	*Dv. thiodismutans*	Tanatar-5 (Kulunda Steppe, Siberia, Russia)	Sorokin et al. (2011c)	NA			
	*Dv. magnus*	Tanatar-5 (Kulunda Steppe, Siberia, Russia)	Sorokin et al. (2011c)	NA			
	*Dv. hydrogenovorans*	Lake Magadi (Kenya)	Zhilina et al. (1997)	NA			
*Desulfonatronospira*	*Dns. thiodismutans*	Kulunda Stepppe (Siberia, Russia)	Sorokin et al. (2008a)	PD	3971	3791	51
	*Dns. delicata*	Wadi Natrun (Egypt)	Sorokin et al. (2008a)	NA			
*Desulfurispira*	*Dsfr. natronophila*	Kulunda Steppe (Siberia, Russia)	Sorokin and Muyzer (2010)	NA			
*Desulfuribacillus*	*Db. alkaliarsenatis*	Kulunda Steppe (Siberia, Russia)	Sorokin et al. (2012d)	NA			
*Anaeroacillus*	*Ab. alkalilacustre*	Lake Khadyn (Tuva Region, Siberia, Russia)	Zavarzina et al. (2009)	NA			
*Cyanospira*	*C. rippkae*	Lake Magadi (Kenya)	Florenzano et al. (1985)	NA			
	*C. capsulata*	Lake Magadi (Kenya)	Florenzano et al. (1985)	NA			
*Clostridium*	*Cl. alkalicellum*	Lake Verkhnee Beloe (Buryatiya, Russia)	Zhilina et al. (2005a, b)	PD	5307	4473	32
*Natronobacillus*	*N. azotofigens*	Kulunda Steppe (Siberia, Russia)	Sorokin et al. (2008e)	NA			
*Tindallia*	*Td. magadii*	Lake Magadi (Kenya)	Kevbrin et al. (1998)	NA			
*Nitrobacter*	*Nb. alkalicus*	Kunkur Steppe (Siberia, Russia)	Sorokin et al. (1998)	NA			
*Halomonas*	*H. mongoliensis*	Lake Dzun-Tukhem-Nur (Mongolia)	Boltyanskaya et al. (2007)	NA			
	*H. kenyensis*	L. Bogoria, L. Nakuru, L. Elmentieta, Crater Lake, L. Magadi (Kenya)	Boltyanskaya et al. (2007)	NA			
*Methanohalophilus (= Methanosalsum)*	*M. zhilinae*	Bosa Lake (Wadi Natrun, Egypt)	Mathrani et al. (1988)	Complete	2138	2083	39
*Methylomicrobium*	*Mm. buryatense*	Lake Khadyn (Siberia, Russia)	Kaluzhnaya et al. (2001); Sorokin et al. (2000)	PD	5067	4530	49
	*Mm. alcaliphilum*	Shara-Nur (Tuva Region, Siberia, Russia)	Kaluzhnaya et al. (2008)	Complete	4668	4083	49
	*Mm. kenyense*	Soda lakes in Kenya	Kaluzhnaya et al. (2008); Sorokin et al. (2000)	NA			
*Methylophaga*	*Mp. alcalica*	Lake Khotontyn (Mongolia	Doronina et al. (2003)	NA			
	*Mp. natronica*	Lake Bulamay (Siberia, Russia)	Doronina et al. (2003)	NA			
*Natroniella*	*Ni. acetigena*	Lake Magadi (Kenya)	Zhilina et al. (1995)	NA			
	*Ni. sulfidigena*	Wadi Natrun (Egypt)/Kulunda Steppe (Siberia, Russia)	Sorokin et al. (2011c)	NA			
*Tindallia*	*Td. californiensis*	Mono Lake (CA, US)	Pikuta et al. (2003b)	NA			
	*Td. magadii*	Lake Magadi (Kenya)	Kevbrin et al. (1998)	NA			
*Natronoincola*	*Ni. histidinovorans*	Lake Magadi (Kenya)	Zhilina et al. (1998)	NA			
*Alkalilimnicola*	*Al. ehrlichii*	Mono Lake (CA, US)	Hoeft et al. (2007)	Complete	3276	2947	68
*Rubribacterium*	*R. polymorphum*	Barguzin River Valley (Siberia, Russia)	Boldareva et al. (2009)	NA			
*Rhodobaca*	*Rh. bogoriensis*	Lake Bogoria, Crater Lake (Kenya)	Milford et al. (2000)	IP	NA	NA	NA
	*Rh. barguzinensis*	Barguzin River Valley (Siberia, Russia)	Boldareva et al. (2008)	NA			
*Rhodovulum*	*Rv. tesquicola*	Sul’fatnoe (Siberia, Russia)	Kompantseva et al. (2012)	NA			
	*Rv. steppense*	Lake Khilganta (Siberia, Russia)	Kompantseva et al. (2010)	NA			
*Spirochaeta*	*Sp. americana*	Mono Lake (CA, US)	Hoover et al. (2003)	NA			
	*Sp. alkalica*	Lake Magadi (Kenya)	Zhilina et al. (1996)	PD	3358	2938	61
	*Sp. africana*	Lake Magadi (Kenya)	Zhilina et al. (1996)	PD	3286	2874	58
	*Sp. asiatica*	Lake Khadyn (Siberia, Russia)	Zhilina et al. (1996)	NA			
*Anoxynatronum*	*An. sibiricum*	Nizhnee Beloe (Siberia, Russia)	Garnova et al. (2003)	NA			
*Alkaliflexus*	*Af. imshenetskii*	Verkhneye Beloye (Buryatiya, Russia)	Zhilina et al. (2004)	PD	4122	3393	43

*NA* not available, *PD* permanent draft, *IP* in progress

## Cultured diversity and their role in biogeochemical cycles

### The carbon cycle

#### Carbon fixation

**Fig. 3:**
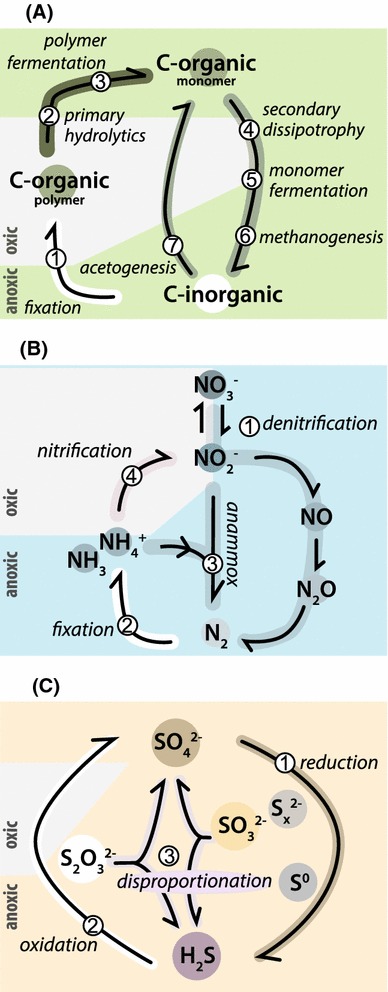
Microbially mediated biogeochemical redox cycles in soda lakes. **a** The carbon cycle, **b** The nitrogen cycle, and **c** the sulfur cycle

Autotrophic primary producers in soda lakes able to fix inorganic CO_2_ into organic polymers include oxygenic and anoxygenic haloalkaliphilic phototrophs and some chemolithoautotrophs (Fig. 3a1). The primary production in most soda lakes is high due to a dense population of haloalkaliphilic cyanobacteria (Melack 1981; Kompantseva et al. 2009). They include unicellular and filamentous heterocystous and non-heterocystous groups. The planktonic cyanobacterial forms, which are dominant in tropical soda lakes in Kenya and Ethiopia (Fig. 1), include the genera *Arthrospira* (*Spirulina*), *Anabaenopsis* and *Cyanospira* (Dubinin et al. 1995; Ballot et al. 2009; Dadheech et al. 2013; Krienitz et al. 2013). Hypersaline soda brines are dominated by the extremely haloalkaliphilic unicellular cyanobacterium ‘*Euhalothece natronophila*’ (Mikhodyuk et al. 2008). Haloalkaliphilic cyanobacteria are most dominant at moderate salinity, whilst at higher salt concentrations only extremely salt-tolerant unicellular green algae, such as *Dunaliella viridis* and *Picocystis salinarium*, can thrive (Gerasimenko et al. 1999; Krienitz et al. 2012; Roesler et al. 2002).

In the south Siberian soda lakes (Kulunda Steppe, Altai) (Fig. 1), where the salinity ranges from 50 to 400 g/l, the most common oxygenic phototrophic communities are represented either by floating aggregates of the green algae *Ctenocladus* and filamentous cyanobacteria or by filamentous cyanobacterial biofilms. The biofilms mainly contain haloalkaliphilic members of the genera *Geitlerinema* and *Nodosilinea* and, occasionally, *Leptolyngbya.* Members from the genera *Arthrospira*, which are dominant in equatorial soda lakes, are virtually absent in this area (O. Samylina, personal communication). At reduced salinity a mass development of heterocystous *Anabaenopsis* had been observed in East-African soda lakes (Krienitz et al. 2013).

Cyanobacteria are traditionally considered as the only diazotrophic component of the oxygenic phototrophic community (Fig. 3b2). However, as they are only moderately salt-tolerant, the identity and mechanisms of primary nitrogen fixation in hypersaline soda lakes remain enigmatic.

Anoxygenic phototrophs, represented by the haloalkaliphilic members of *Chromatiales* (*Thiorhodospira*, *Thiorhodovibrio*) at moderate salinity and *Ectothiorhodospiracea* (*Ectothiorhodospira*/*Halorhodospira*) at high salinity, also contribute to the primary production in soda lakes (Gorlenko 2007; Kompantseva et al. 2009). ‘Secondary’ primary producers represented by aerobic chemolithoautotrophic bacteria also contribute to inorganic carbon fixation in soda lakes. Haloalkaliphilic representatives of nitrifying, sulfur-oxidizing, H_2_-oxidizing, and carboxydotrophic bacteria have recently been isolated from soda lakes and characterized (Sorokin and Kuenen 2005; Grant and Sorokin 2011).

#### Heterotrophic carbon utilization

The heterotrophic bacteria, responsible for the primary degradation of organic matter produced by the autotrophic bacteria, include aerobes and fermentative anaerobes, which in turn, are composed of two subgroups: the hydrolytics (Fig. 3a2), which degrade polymers, and the secondary heterotrophs (‘dissipotrophs’) (Figs. 3a4) that utilize the resulting monomers.

Aerobic hydrolytics, which produce alkali-stable hydrolases, have been the focus of many studies in the past, because of the high application potential of their enzymes in industry (Horikoshi 2004, 2006). However, only few of the known isolates were recovered from soda lakes. They mostly include aerobic Firmicutes, such as species within the genus *Bacillus* with various glycosidase activities and several Actinobacteria, such as *Cellulomonas* and *Dietzia*, and Gammaproteobacteria, such as the amylolytic *Alkalimonas* (Grant and Sorokin 2011). Recently, it was shown that aerobic haloalkaliphilic Actinobacteria and Gammaproteobacteria from the genus *Marinimicrobium* from soda lakes and soda soils can utilize chitin as growth substrate (Sorokin et al. 2012a). So far, only a single pure culture of an anaerobic low salt-tolerant cellulolytic bacterium has been recovered from a soda lake represented by *Clostridium alkalicellum* (Zhilina et al. 2005a). Two recently described fermentative anaerobic haloalkaliphiles from soda lakes can use pectin as substrate either at moderate (*Natronoflexus pectinovorans* from the Bacteriodetes) or high salt concentration (*Natronovirga* from the *Clostridiales*) (Sorokin et al. 2011a, 2012b) (Fig. 3a3). Additionally, two deep lineages of fermentative haloalkaliphilic bacteria specialized to exclusively utilize chitin as growth substrate were isolated from soda lakes. Both groups belong to the phylum TG3, which, until now, only included uncultured bacteria (Sorokin et al. 2012a). The high salt-tolerant group has recently been described as *Chitinivibrio alkaliphilus* (Sorokin et al. 2014a).

Secondary (‘dissipotrophic’) heterotrophs, i.e., those that utilize monomeric organic compounds such as sugars, amino acids, organic acids, and alcohols are among the best represented groups of haloalkaliphiles isolated so far from soda lakes (Fig. 3a4). Among the aerobes, haloalkaliphilic members of the genus *Halomonas* from the Gammaproteobacteria, *Bacillus* from the Firmicutes, and Actinobacteria are the most abundant (Duckworth et al. 1996; Grant and Sorokin 2011). Soda lake fermentative anaerobes are dominated by haloalkaliphilic representatives of Clostridia, such as members of the genera *Anoxynatronum*, *Anaerovirgula*, *Alkaliphilus*, *Natranaerobius*, *Natranaerobaculum*, and certain species of *Anaerobranca*, *Spirochaeta*, and *Anaerobacillus* (Zavarzin et al. 1999; Zavarzin and Zhilina 2000; Bowers et al. 2009; Grant and Sorokin 2011; Mesbah and Wiegel 2012) (Fig. 3a5). Among the secondary anaerobes, which function during the last stage of organic carbon degradation, homoacetogens (Fig. 3a7) and methanogens (Fig. 3a6) represent the least studied functional groups of soda lake microbial communities. The genera *Tindallia*, *Natronincola* (*Clostridiales*), and *Natroniella acetogena* (*Halanaerobiales*) represent heterotrophic fermentative haloalkaliphilic acetogens, utilizing amino acids and alcohols as substrates (Kevbrin et al. 1998; Zhilina et al. 1995, 1998). Hydrogenotrophic acetogens in soda lakes have only recently been discovered. The only culturable organism is represented by a novel, extremely salt-tolerant haloalkaliphilic member of the *Halanaerobiales* described as *Fuchsiella alkaliacetigena* (Zhilina et al. 2012).

#### Methane cycle

The methane cycle has been explored in soda lakes as an important part of the microbial carbon cycle. Substantial efforts have been made to detect methanogenic activity in anaerobic sediments from North American and Central Asian soda lakes (Fig. 1). The results clearly demonstrated a dominance of methylotrophic methanogenesis and absence of acetoclastic processes, while the results concerning hydrogenotrophic methanogenesis were inconclusive (Oremland and Miller 1993; Namsaraev et al. 1999; Sorokin et al. 2004a; Nolla-Ardèvol et al. 2012). Some of the key haloalkaliphilic players in soda lake methanogenesis have been isolated in pure culture and described, including two groups of methylotrophs, such as *Methanolobus taylorii* (moderate salinity) and *Methanosalsum zhilinae* (high salinity), and a highly salt-tolerant lithotroph *Methanocalculus natronophilus* (Mathrani et al. 1988; Oremland and Boone 1994; Kevbrin et al. 1997; Zhilina et al. 2013).

Aerobic methanotrophs in soda lakes are dominated by low salt-tolerant alkaliphiles from the Gammaproteobacterial genus *Methylomicrobium* (Sorokin et al. 2000; Trotsenko and Khmelenina 2002). Assuming that methanotrophic alkaliphiles cannot grow at salinities above 1.5 M of total Na^+^, while methane production still occurs at these high salinity values, the methane cycle in hypersaline soda lakes may be incomplete, similar as in hypersaline chloride–sulfate lakes (Conrad et al. 1995).

### The nitrogen cycle

Denitrification in soda lakes is performed by heterotrophs dominated by extremely salt-tolerant alkaliphilic representatives of the genus *Halomonas* (Shapovalova et al. 2009) and by several facultative anaerobic lithotrophs, such as representatives of the genus *Thioalkalivibrio* (see below) and the *Alkalilimnicola*–*Alkalispirillum* group of the Gammaproteobacteria (Sorokin et al. 2006; Hoeft et al. 2007) (Fig. 3b1). Whether dissimilatory ammonification competes with denitrification in soda lakes has not yet been resolved. So far, the alkaliphiles with this metabolism have only been found in bioreactors operating at high pH, i.e., *Desulfurispirillum alkaliphilum* from the phylum Chrysiogenetes (Sorokin et al. 2007b) and *Sulfurospirillum alkalitolerans* from the Epsilonproteobacteria (Sorokin et al. 2013a).

Heterotrophic anaerobic fermentative haloalkaliphiles actively fix nitrogen in soda lakes and soda soils (Sorokin et al. 2008c) (Fig. 3b2). These organisms are represented by two groups of the Firmicutes: a moderate salt-tolerant *Anaerobacillus diazotrophicus (*reclassified from *Bacillus alkalidiazotrophicus)* (Sorokin et al. 2008d) and a highly salt-tolerant *Natronobacillus azotifigans* (Sorokin et al. 2008e). Furthermore, the microbial activity and presence of the *nifH* gene, encoding a nitrogenase, have also been detected in two other soda lake anaerobes: the iron-reducing *Geoalkalibacter ferrihydriticus* (Zavarzina et al. 2006) and the cellulolytic *Clostridium alkalicellulosi* (Zhilina et al. 2005a). Additionally, the *nifH* gene has been detected in several soda lake anoxygenic phototroph representatives (Tourova et al. 2007), which indicates that anoxygenic phototrophs may also contribute to nitrogen fixation. Not much research has been conducted on nitrogen fixation in soda lakes at oxic conditions. However, a major suspect is a group of heterocystous low salt-tolerant alkaliphilic cyanobacteria from the *Anabaena* group (*Anabaenopsis* and *Nodularia*) (O. Samylina, personal communication). Nitrogen fixation activity has been documented for aggregates of filamentous non-heterocystous *Phormidium*-like cyanobacteria and green algae belonging to the *Ctenocladus* in the oxic littoral zone of Mono Lake (Oremland 1990). However, it was not clear whether the phototrophic or the heterotrophic bacteria were responsible for the observed diazotrophy.

The ammonium produced during nitrogen fixation in soda lakes can be oxidized to nitrate via nitrite by haloalkaliphilic nitrifiers (Fig. 3b4). In soda lakes and soda soils ammonium oxidation to nitrite is performed by an extremely alkali-tolerant subpopulation of *Nitrosomonas halophila*, whilst nitrite oxidation can be performed by the moderately alkali-tolerant *Nitrobacter alkalicus* (Sorokin and Kuenen 2005). Since the maximum salt concentration for nitrification in soda lakes is 1 M of total Na^+^ (Sorokin 1998), the nitrogen cycle is inhibited in hypersaline soda lakes. In addition, the NH_3_/NH_4_^+^ equilibrium at high pH favors the formation of toxic NH_3_ and, therefore, causes potential N-loss from the ecosystem (Tindall 1988; Sorokin and Kuenen 2005). Therefore, the nitrogen cycle in soda lakes, especially in hypersaline ones, may depend on an externally supplied source of NO_*x*_^−^.

### The sulfur cycle

#### Sulfidogenesis

The dissimilatory reduction of oxidized sulfur compounds such as sulfate, sulfite, thiosulfate, and sulfur, resulting in sulfide production (sulfidogenesis) are important biogeochemical processes within soda lakes (Sorokin et al. 2010a, 2011b) (Fig. 3c1). Several obligatory anaerobic and obligatory haloalkaliphilic bacteria can perform these reactions. Members of the deltaproteobacterial genera *Desulfonatronum*, *Desulfonatronovibrio*, and *Desulfonatronospira* represent lithotrophic sulfate-reducing bacteria (SRB) in soda lakes (Sorokin et al. 2011c). They can grow either as typical SRB by oxidizing hydrogen, formate or short-chain organic compounds as electron donor, and sulfate, thiosulfate or sulfite as electron acceptor, or they can obtain energy by thiosulfate or sulfite disproportionation (Sorokin et al. 2008a, 2011b). Heterotrophic SRB in soda lakes belong to the group of incomplete oxidizers, utilizing either propionate (*Desulfobulbus alkaliphilus*) or butyrate (*Desulfobotulus alkaliphilus*) as e-donor/C-source with sulfate or thiosulfate as e-acceptor and forming acetate as a final product (Sorokin et al. 2010b; Sorokin et al. 2012d). So far, only a single haloalkaliphilic SRB, described as *Desulfonatronobacter acidivorans*, has been found in soda lakes, which belongs to the complete oxidizers (Sorokin et al. 2012c). It can oxidize several volatile fatty acids (VFA) completely to CO_2_ with sulfate or thiosulfate as e-acceptor, but cannot utilize externally provided acetate. Haloalkaliphilic syntrophic associations of reverse acetogenic *Clostridiales* members and lithotrophic SRB drive acetate oxidation in soda lakes under sulfate-reducing conditions. At low salt concentrations the association includes “*Candidatus* Contubernalis alkalaceticum” and *Desulfonatronum cooperativum* (Zhilina et al. 2005b), whilst at extremely high salt concentrations the association contained “*Candidatus* Syntrophonatronum acetioxidans” and *Desulfonatronospira* sp. (Sorokin et al. 2014b).

Elemental sulfur reduction in soda lakes is probably not performed by SRB, since none of the pure cultures of haloalkaliphilic SRB can grow with sulfur as e-acceptor. Instead, three different lineages of obligatory anaerobic haloalkaliphiles are implicated in sulfur reduction. In all three, the actual e-acceptor is not sulfur itself, but polysulfide (S_*x*_^2−^) forming abiotically at high pH from sulfur and sulfide. The first, *Desulfurispira natronophila*, belongs to the phylum Chrysiogenetes (Sorokin and Muyzer 2010). The second group of sulfur/polysulfide-respiring haloalkaliphiles, belongs to the Firmicutes and was isolated from soda lakes with formate as e-donor. A moderately salt-tolerant representative is described as *Desulfuribacillus alkaliarsenatis* (Sorokin et al. 2012d). Apart from sulfur, it can also use arsenate and thiosulfate as e-acceptors. Third, at saturated soda concentrations, microbial-mediated sulfur reduction can also be performed by *Natroniella sulfidigena*, which belongs to the *Halanaerobiales*. It can use acetate, H_2,_ and formate as e-donors for sulfur/polysulfide-dependent respiration (Sorokin et al. 2011d).

#### Elemental sulfur disproportionation

Two anaerobic low salt-tolerant alkaliphilic anaerobes from soda lakes, *Dethiobacter alkaliphilus* and *Desulfurivibrio alkaliphilus*, originally described as sulfur and thiosulfate reducers (Sorokin et al. 2008b), have the capability to grow chemolithoautotrophically by sulfur or polysulfide disproportionation (Fig. 3c3). Remarkably, they are the first alkaliphiles with such a physiology, and in contrast to the neutrophilic sulfur disproportionators, they do not require the presence of ferric iron to precipitate toxic sulfide compounds (Poser et al. 2013).

#### Sulfur-oxidizers

Sulfide produced by sulfidogens can be oxidized to elemental sulfur or sulfate by phototrophic and chemotrophic sulfur oxidizing bacteria (SOB) (Fig. 3c2). In soda lakes, the former are dominated by anoxygenic purple sulfur bacteria, including haloalkaliphilic members of the genera *Ectothiorhodospira* and *Halorhodospira* at high salinity, and members of the genera *Thiorhodospira*, *Thioalkalicoccus*, and *Ectothiorhodosinus* at low salinity (Imhoff and Trueper 1981; Gorlenko 2007). The chemotrophic SOB in soda lakes belong to 4 genera of the haloalkaliphilic Gammaproteobacteria: the genera *Thioalkalimicrobium* and *Thioalkalispira* are moderate salt-tolerant aerobic alkaliphiles, while the genera *Thioalkalivibrio* and *Thioalkalibacter* can grow in salt concentrations reaching saturation (Sorokin et al. 2013b). They are obligate autotrophs and utilize reduced sulfur compounds, including sulfide, polysulfide, thiosulfate, polythionates, and elemental sulfur as e-donor (Sorokin et al. 2001b, c, 2002b, 2003; Banciu et al. 2004) The genus *Thioalkalivibrio* is the most metabolically flexible and tolerates a wide range of salinity values. Several *Thioalkalivibrio* species have the ability to grow anaerobically with NO_*x*_^−^ as e-acceptors (Fig. 3b1), such as *Tv. denitrificans* (Sorokin et al. 2001b), *Tv. nitratireducens* (Sorokin et al. 2003), and *Tv. thiocyanodenitrificans* (Sorokin et al. 2004b).

Other *Thioalkalivibrio* species such as *Tv. thiocyanoxidans*, *Tv. paradoxus*, and *Tv. thiocyanodenitrificans*, are capable of growth using thiocyanate as the sole energy, sulfur, and nitrogen source (Sorokin et al. 2001c, 2002b, 2004b). The first two species degrade thiocyanate primarily to cyanate and were the first SOB cultures for which the cyanate pathway of primary thiocyanate degradation has been shown.

## Prokaryotic diversity, activity, and community structure identified by cultivation-independent approaches

It is well recognized that from the majority of the microorganisms in nature, cultured isolates are yet to be obtained. Alternative cultivation-independent approaches, especially those based on the characterization of DNA, have proven to be very useful in expanding the known diversity of the microbial communities thriving under the extreme conditions of high salinity and high pH. Grant et al. (1999) were the first to use molecular methods to study the archaeal diversity of saturated alkaline brines in Lake Magadi (Kenya, Africa) (Fig. 1). Thereafter, the presence of novel prokaryotic phylotypes in various soda lakes was shown by cloning and/or denaturing gradient gel electrophoresis (DGGE) of 16S rRNA gene fragments (Ochsenreiter et al. 2002; Rees et al. 2004; Ma et al. 2004; Mesbah et al. 2007). More recently, next-generation sequencing of PCR-amplified regions of the 16S rRNA gene and reversed transcribed mRNA have been used (Lanzen et al. 2013).

Cultivation-independent approaches have also greatly improved our understanding of the overall microbial community structure and functioning in soda lakes, which seems to be governed by the prevailing salt concentrations. There is some evidence that hypersaline soda lake brines (total salinity >250 g/L) harbor similar microbial communities to hypersaline solar saltern brines of neutral pH. The latter are characterized by a low diversity dominated by a few extremely halophilic archaea, belonging to the class Halobacteria within the phylum Euryarchaeota (Rodriguez-Valera et al. 1985; Oren 1994; Casamayor et al. 2002; Ghai et al. 2011). It is conceivable that hypersaline soda lake brines may also be dominated by such archaea, as evidenced by the failure to amplify bacterial 16S rRNA genes from the soda brines (Grant et al. 1999) and the clear dominance of euryarchaeal sequences in 16S rRNA gene libraries (Grant et al. 1999; Ochsenreiter et al. 2002; Mesbah et al. 2007). The latter sequences share a high similarity with members from the family *Halobacteriaceae* (class *Halobacteria*; Ochsenreiter et al. 2002; Mesbah et al. 2007) and from halophilic members of the order *Methanosarcinales *(class *Methanomicrobia*) (Mesbah et al. 2007).

Moderately saline soda lake brines (total salinity between 50 and 250 g/L) harbor more diverse microbial communities than hypersaline environments and the community composition is affected by lake stratification and prevailing oxygen concentrations (Dimitriu et al. 2008; Carini and Joye 2008). The total bacterial and archaeal diversity in low saline lakes (total salinity between 35 and 50 g/L) can be as high as that in fresh water lakes (Lanzen et al. 2013). Several studies on the bacterioplankton from low and moderate saline soda lakes showed the dominant presence of Alphaproteobacteria (mostly from the family *Rhodobacteraceae*) and Gammaproteobacteria (including the genera *Halomonas* and *Thioalkalivibrio*), Firmicutes (aerobic *Bacillus*, anaerobic *Clostridia)*, Bacteroidetes (*Cytophaga*, *Flexibacter*, *Flavobacterium*, *Bacteroides*, *Salinibacter*), the cyanobacterial genera *Arthrospira* and *Anabaenopsis*, and several purple phototrophic bacteria belonging to the families of *Ectothiorhodospiraceae*, *Chromatiaceae* and *Rhodobacteraceae* (Humayoun et al. 2003; Dimitriu et al. 2008; Mesbah et al. 2007; Pagaling et al. 2009; Lanzen et al. 2013; Dadheech et al. 2013; Asao et al. 2011).

The salt concentration in the sedimentary pore water of soda lakes also has a strong influence on the in situ microbial community composition (Mesbah et al. 2007) and negatively affects the diversity (Kulp et al. 2007; Foti et al. 2008). In addition, some of the fundamental biogeochemical cycles are hampered through the inhibition of key catabolic transformations, such as denitrification, sulfate reduction, and methanogenesis (Kulp et al. 2007; Sorokin et al. 2010a). Nevertheless, bacterial 16S rRNA from clone libraries and DGGE bands from moderate and hypersaline soda lake sediments was found to be relatively diverse, including various Alphaproteobacteria from the order *Rhodobacterales* or related to the genus *Brevundimonas*; Firmicutes (mainly *Clostridia*), Gammaproteobacteria, Bacteroidetes, Betaproteobacteria (genera *Alcaligenes* and *Comamonas*), Deltaproteobacteria (orders *Desulfovibrionales* and *Desulfobacterales*), Actinobacteria (moderate salinities), and benthic cyanobacteria (Mesbah et al. 2007; Ma et al. 2004; Foti et al. 2008; Dimitriu et al. 2008; Kulp et al. 2006).

Molecular studies targeting functional genes are necessary to identify possible microbial-mediated processes within the biogeochemical element cycles (Fig. 3). Giri et al. (2004) were the first to use *cbbL/M* genes, encoding the large subunit of RuBisCo form I/II (Watson and Tabita 1997), as a functional and phylogenetic marker for autotrophs in soda lakes. They studied the distribution of these genes along a redox gradient in the sediment of Mono Lake (USA).

To study the diversity of autotrophic bacteria in soda lake sediments from the Kulunda Steppe (Siberia, Russia) and Wadi Natrun (Egypt) (Fig. 1), Kovaleva and colleagues (Kovaleva et al. 2011) used *aclB*, which encodes the large subunit of ATP citrate lyase part of the reverse Krebs cycle, in addition to *cbbL/M* (Campbell et al. 2003). Overall, most autotrophs in the studied soda lake sediments use the Calvin–Benson–Bassham cycle for inorganic carbon fixation, with RuBisCO form I as the dominant and most diverse type. More specifically, the autotrophs in the sediments of hypersaline soda lakes were primarily composed of cyanobacteria and haloalkaliphilic SOB from the family *Ectothiorhodospiraceae* (class Gammaproteobacteria, order *Chromatiales*), including the chemolithotrophic genus *Thioalkalivibrio* and the phototrophic genus *Halorhodospira* (Giri et al. 2004; Kovaleva et al. 2011). In the less saline lakes, distinct novel lineages of anoxygenic phototrophs with RuBisCO form I within the order *Chromatiales* were found (Kovaleva et al. 2011). Autotrophic nitrification in Mono Lake was studied via bacterial and archaeal *amoA* and 16S rRNA gene libraries (Carini and Joye 2008). Samples were taken after an extended period of meromixis during which significant nitrification was measured and the mixoliminion was presumed to have become chronically N-deprived (Joye et al. 1999; Carini and Joye 2008). Ammonia monooxygenase catalyzes the first step in aerobe ammonium oxidation by autotrophic nitrifiers, and *amoA*, encoding its active-site polypeptide, is frequently used as a functional marker (Junier et al. 2010).

Sequences obtained from ammonia-oxidizing bacteria (AOB) were most closely related to halo- and/or alkali-tolerant *Nitrosomonas*-like sequences. Additionally, FISH analysis revealed the presence of Crenarchaeota and the correlation of nitrification rates with crenarchaeal numbers. Although no archaeal *amoA* sequences were detected, it cannot be ruled out that ammonia-oxidizing archaea (AOA) contribute significantly to nitrification in Mono Lake (Carini and Joye 2008). Key functional genes of dissimilatory SRB are *dsr*AB, which encodes the α- and β-subunits of a dissimilatory sulfite reductase, and *aps*A, which encodes the α-subunit of an APS reductase (Wagner et al. 2005). Two independent studies focusing on these genes in sediment samples and enrichment cultures from Mono Lake (USA; Scholten et al. 2005) and soda lakes in Siberia (Russia; Foti et al. 2007) revealed novel clusters of SRB affiliated to the deltaproteobacterial order *Desulfovibrionales* and the family *Desulfobacteraceae* within the order *Desulfobacterales*. The latter comprises all of the known SRB that oxidize acetate completely during sulfate reduction. In combination with high *dsrB* copy numbers per cell and sulfate reduction rates encountered even in soda lakes with more than 475 g/L, Foti et al. (2007) challenged an earlier hypothesis, specifically for the case of soda lakes, that complete carbon oxidizers could only grow at salt concentrations below 150 g/L (Oren, 1999 and Oren 2011).So far, no acetate-oxidizing SRB have been isolated from soda lakes, even at low salinity.

The oxidative part of the sedimentary sulfur cycle was also studied in various soda lakes from Siberia and Egypt (Tourova et al. 2013) by targeting *soxB*, which encodes an indispensible sulfate thiohydrolase in the *Sox* pathway proposed for the oxidation of thiosulfate in SOB (Ghosh and Dam 2009). The majority of detected SOB sequences belonged to autotrophic Gammaproteobacteria, including the genus *Thioalkalivibrio* from which already many cultured isolates have been obtained. Interestingly, uncultured putative heterotrophic SOB from the Gamma-and Alphaproteobacterial classes have been found by comparing *soxB* clone libraries (Tourova et al. 2013) with earlier constructed *cbbL/M* genes from the same sediment samples (Kovaleva et al. 2011).

A very effective technique to study microbial activity is the use of stable isotope probing (SIP; Dumont and Murrell 2005). Lin et al. (2004) used this approach to identify active methanotrophs in sediments of a low saline Transbaikal soda lake. By targeting both 16S rRNA genes as well as genes encoding pmoA and mmoX, key enzymes in the aerobic methane oxidation pathway (McDonald et al. 2008), they found that the type 1 methanotrophs, belonging to the gammaproteobacterial genera *Methylomicrobium* and *Methylobacter*, were the main methane oxidizers. Active aerobic methane oxidation, as well as archaeal ammonium oxidation (ammonium oxidation to nitrite) and denitrification (nitrite reduction to nitrous oxide) in the water column of two low saline Ethiopian soda lakes was also shown by the successful amplification of mRNA for particulate methane monooxygenase (lake Beseka), ammonia monooxygenase (*amoA*) and nitrite reductase (*nirK*; lake Arenguadi; Lanzen et al. 2013).

## Cellular adaptations to high salt concentrations and high pH values

**Fig. 4:**
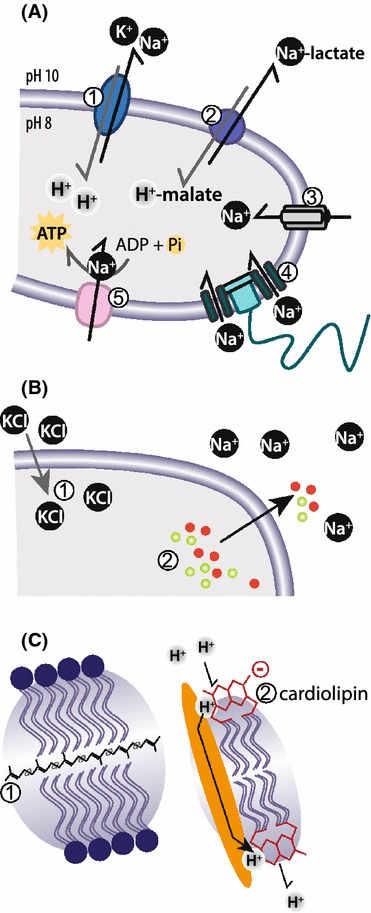
Proposed adaptation strategies to the extreme haloalkaline environment. **a** Bioenergetic adaptations: *1* Electrogenic proton antiporters with Na^+^ or K^+^. *2* Electroneutral antiporters. *3* Voltage-gated Na^+^ channel. *4* Na^+^-dependent flagella. *5* Na^+^ ATPase. **b** Osmoprotectants retain the osmotic pressure within the cell via the *1* Salt in cytoplasm strategy or *2* the synthesis or accumulation of osmoprotectants. **c** Structural membrane adaptations to survive the extreme haloalkaline conditions: *1* squalene or *2* cardiolipins

Haloalkaliphilic bacteria have developed essential strategies to adapt to the extreme haloalkaline conditions in soda lakes (Padan et al. 2005; Slonczewski et al. 2009). Although not much is known about the genetics of these adaptations, some bioenergetic and structural adjustments that ensure the maintenance of an intracellular neutral pH and osmotic pressure have mainly been described in the species *Bacillus halodurans* C125 and *B. pseudofirmus* OF4 (Kitada et al. 1994; Ito et al. 2004; Janto et al. 2011). The membrane structure of alkaliphiles remains stable over a wide range of pH and salinity values and is poorly permeable to protons and sodium ions (van de Vossenberg et al. 1999). Therefore, these microbes use transporter proteins to mediate the transmembrane pH gradient (ΔpH) and electric potential (ΔΨ). The intracellular pH is regulated by several transporter mechanisms, one of which is mediated by electrogenic antiporters that import protons to the cytoplasm, whilst exporting a counterbalancing monovalent cation: Na^+^ or K^+^ (Ito et al. 1997; Kitada et al. 1994; Hunte et al. 2005; Mesbah et al. 2009; Muyzer et al. 2011, 2012) (Fig. 4a1). It has been shown that *Escherichia coli* cells require a Na^+^:H^+^ exchange ratio of at least 1:2 to support growth in alkaline environments (Pinner et al. 1993); however, the coupling stoichiometry of alkaliphiles isolated from soda lakes has so far not been determined yet. An alternative electro-neutral proton transporter has been described in *B. subtilis*. It functions in carrying proton-bound malate into the cell whilst cytoplasmic sodium-bound lactate is exported (Wei et al. 2000) (Fig. 4a2). Whether this transporter plays a potential role in the pH homeostasis of haloalkaliphiles remains to be elucidated. Another group of sodium transporters that are thought to play a role in pH homeostasis and also in motility and chemotaxis under alkaline conditions is the voltage-gated Na^+^ channel, encoded by *ncbA* in *B. pseudofirmus* OF4, (Ito et al. 2004; Fujinami et al. 2007) (Fig. 4a3). These channels can be co-localized with methylated chemotaxis proteins (Fujinami et al. 2007). Chemotaxis is also mediated through motility, which is sodium dependent in *B. pseudofirmus* (Ito et al. 2005; Fujinami et al. 2009) (Fig. 4a4). In the absence of sodium, potassium or rubidium can replace the role of sodium (Terahara et al. 2012). Last, an ATPase driven by a sodium motive force instead of a proton motive force has been identified in extremely salt-tolerant alkaliphilic clostridia *Natranaerobius* (Mesbah and Wiegel 2011) (Fig. 4a5). Although such a protein has not yet been discovered in haloalkaliphiles, it is conceivable that they may possess a similar mechanism, which utilizes the excess sodium and maintains a high transmembrane electric potential.

The high salinity in soda lakes also causes a high degree of osmotic stress to haloalkaliphiles, requiring them to synthesize osmoprotectants. In order to retain turgor pressure, halophilic microorganisms are known to either use the “salt in cytoplasm” strategy, where intracellular KCl concentrations are kept higher than the extracellular concentrations (Fig. 4b1), or to synthesize or accumulate compatible solutes during which high concentrations of neutral soluble organic molecules are stored in the cytoplasm (Rössler and Müller 2001) (Fig. 4b2). The compounds glycine betaine, glutamine, proline, ectoine, and hydroxyectoine have been found to play major roles as compatible solutes in bacteria (Grammann et al. 2002; Banciu et al. 2005; Hoffmann et al. 2012; Sorokin et al. 2013b). Extremely halo(alkali)philic Euryarchaeota predominantly utilize K^+^ as an osmotic regulator (Oren 1999, 2011). These osmolytes do not play an active role in the metabolism of the cell, but are pivotal to the cellular volume and homeostasis (Levy-Sakin et al. 2014), and have been shown to stabilize membrane protein structures (Burg and Ferraris 2008; Roychoudhury et al. 2013). Although the “salt out” strategy of osmotic regulation is energetically more expensive than the “salt in” strategy, it allows microorganisms with a highly efficient energy metabolism to survive over larger salinity gradients (Oren 2011).

Structural adjustments within the cell membrane of haloalkaliphilic prokaryotes include an increased level of the neutral lipid squalene and the polar lipid cardiolipin in the phospholipid bilayer (Angelini et al. 2012). Squalene has also been found in the lipid bilayer membrane of the bacterium *Thioalkalivibrio versutus* strain ALJ 15. Squalene functions in combination with cyclopropane fatty acids in the maintenance of their cellular membrane and might prevent proton leakage (Banciu et al. 2005). Squalene is physically positioned in the center of the membrane, perpendicular to the two lipid layers that comprise the membrane (Hauss et al. 2002) (Fig. 4c1). Another class of lipids found in bacterial membranes (*Thioalkalivibrio)* is cardiolipin (Banciu et al. 2005), whose negative charge prevents protons from diffusing away from the cells (Haines and Dencher 2002) (Fig. 4c2). The membrane lipids of extremely halophilic Euryarchaea contain a large amount of diacidic phospholipids (Tenchov et al. 2006). Some extremely halophilic prokaryotes, such as memebrs of the *Halobacteriaceae* and *Salinibacter*, have membrane surface layers that are strongly enriched in acidic amino acids (Oren 2013). The proteins may enable the bacteria to influence the co-ordination of water molecules on their surface membranes facilitating their solubility at higher salt concentrations (Talon et al. 2014). Ecophysiological experiments followed by transcriptome and proteome analyses will offer an opportunity to provide additional insight into the molecular mechanisms by which these organisms adapt to extreme conditions of high pH and salinity.

## Perspectives

Traditionally, studies on microbial communities were restricted to a few cultured isolates, whilst modern high-throughput techniques now allow the study of microbial community composition as a whole. Although a considerable number of cultured isolates has already been obtained from soda lakes, culture-independent methods have uncovered a much more diverse microbial community. Future studies should attempt to isolate members of the uncultured community (Alain and Querellou 2009). Meta-omic approaches might help to facilitate the isolation of microbes by providing insight into potential metabolisms, such as for the isolation of the ammonium-oxidizing archaeon *Nitrosopumilus maritimus* (Könneke et al. 2005). Amplicon sequencing of 16S rRNA gene fragments followed by co-occurrence analysis might shed light onto the different interactions of the community members (Barberán et al. 2012). The detection of functional genes and their transcripts might reveal additional diversity and potential niche differentiation.

Metagenomics can be applied to obtain a high-resolution genetic inventory of the microbial community in soda lakes. Such a genetic inventory can be used to explore the overall metabolic capacity of the prokaryotic soda lake communities. Other high-throughput techniques monitor community-wide levels of gene-expression (meta-transcriptomics; Carvalhais et al. 2012), protein abundance (meta-proteomics; Verberkmoes et al. 2009), and metabolite abundance (meta-metabolomics), thereby generating data to facilitate systems biology approaches.

Several metabolic processes might be present in soda lakes but have not yet been detected, such as anaerobic methane oxidation and anaerobic ammonium-oxidation. Furthermore, the importance of anaerobic polymer degradation in sediments is not well understood and the contribution of anoxygenic photosynthesis to primary production might be underestimated. Nitrogen fixation at hypersaline conditions is suspected to be limited to diazotrophic anoxygenic phototrophs and heterotrophs, but a comprehensive study targeting *nifH* and *nifD* genes in soda lakes, and distinguishing between the contribution of heterotrophs and primary producing phototrophs, is still lacking. Single cell techniques, like FISH-NanoSIMS (Dekas and Orphan 2011), may provide clear answers here. The effect of salinity on other reactions in the nitrogen cycle, such as nitrification, could be confirmed by focusing on the detection and quantification of *amoA* genes and their transcripts.

In conclusion, several questions regarding biogeochemical cycles in soda lakes are still open. To obtain a more comprehensive insight into the microbial diversity of soda lakes, its role in biogeochemical cycles and the molecular mechanisms by which the microorganisms adapt to the extreme environmental conditions, we have to study these habitats with a systems biology approach in which we combine novel isolation methods with state-of-the-art meta-omics techniques, and eventually with mathematical modeling to predict the response of cells and communities to environmental stimuli and to infer the interactions of co-existing populations.
